# Quantifying Greenhouse Gas Emissions and Carbon Footprint of Sheep Production Using the IPCC Tier 2 Approach

**DOI:** 10.3390/ani16071099

**Published:** 2026-04-02

**Authors:** Busra Yayli, Ilker Kilic

**Affiliations:** Department of Biosystems Engineering, Faculty of Agriculture, Bursa Uludag University, 16059 Bursa, Türkiye; ikilic@uludag.edu.tr

**Keywords:** carbon footprint, greenhouse gas emissions, emission factors, manure management, methane, small ruminants, Tier 2

## Abstract

Livestock production significantly contributes to greenhouse gas (GHG) emissions, particularly methane (CH_4_) and nitrous oxide (N_2_O) originating from enteric fermentation and manure management. This study quantified the GHG emissions and cumulative carbon footprint of four commercial sheep farms (SF1, SF2, SF3, and SF4) in the Bursa region of Türkiye, with flock sizes of 200, 500, 150, and 800 adult Merino sheep (mature ewes and breeding rams), respectively. Using the IPCC Tier 2 methodology, the biogenic carbon footprint was estimated at 15.6 kg CO_2_-eq per kg of boneless sheep meat. However, when indirect inputs were included, the cumulative carbon footprint reached 28.8 kg CO_2_-eq for ewes and 32.3 kg CO_2_-eq for breeding rams. These results indicate that indirect emissions from feed production account for the primary environmental load (49.8%), while on-farm energy-related emissions represent a minor portion (0.3%) of the total impact. The results demonstrate that while enteric fermentation (32.5%) remains a critical biological factor, the environmental burden of the feed supply chain is equally significant in intensive systems. These findings highlight that excluding indirect inputs leads to a substantial underestimation of the climate impact, suggesting that mitigation strategies must integrate nutritional optimization with enteric methane reduction to decarbonize sheep production effectively.

## Simple Summary

Sheep production contributes to greenhouse gas (GHG) emissions, primarily through the release of methane (CH_4_), nitrous oxide (N_2_O), and carbon dioxide (CO_2_). This study examined the total climate impact of four sheep farms in Türkiye, focusing on enteric fermentation, manure management, emissions from feed production, and on-farm energy use. When all factors were integrated, the cumulative carbon footprint was found to be 28.8 kg CO_2_-eq for ewes and 32.3 kg CO_2_-eq for breeding rams per kg of meat. These findings provide a comprehensive baseline, emphasizing that mitigating the environmental impact of sheep farming requires strategies that address both animal physiology and the sustainability of feed supply chains.

## 1. Introduction

Climate change has emerged as a critical global challenge driven by rising atmospheric concentrations of greenhouse gases (GHGs), largely attributable to anthropogenic activities. Agriculture and land-use practices are recognized as major sources of GHG emissions, with the livestock sector representing a substantial contributor among anthropogenic activities [1]. Livestock production systems are significant sources of GHG emissions, notably methane (CH_4_), carbon dioxide (CO_2_), and nitrous oxide (N_2_O) [2,3,4]. Although emitted in lower quantities, CH_4_ and N_2_O exhibit considerably higher global warming potentials than CO_2_, underscoring the imperative to mitigate emissions from ruminant systems. According to the IPCC Sixth Assessment Report, substantial reductions in these gases are essential to prevent global temperatures from exceeding the 1.5–2.0 °C threshold during the 21st century [5].

Within sheep production systems, CH_4_ from enteric fermentation is the predominant emission source, while N_2_O from manure management and excreta deposition represents the second-largest contributor [6,7]. Although cattle dominate ruminant emissions, enteric fermentation in small ruminants contributes approximately 0.5 Gt CO_2_-eq year^−1^ globally [8]. Quantifying these emissions using the carbon footprint (CF) metric is essential, as it enables standardized comparisons of environmental impacts across diverse production systems and meat types per unit of product [9].

Türkiye possesses extensive agricultural resources and a robust livestock sector [10]. As of 2024, the national inventory included approximately 54.9 million small ruminants, with sheep accounting for nearly 44 million head [11]. Türkiye’s sheep population significantly exceeds that of all European Union member states, surpassing major producers such as Spain, Romania, and Greece by severalfold [12]. Among the predominant breeds, Merino sheep—including Karacabey, Malya, and Anatolian hybrids—are distinguished by their high adaptability to marginal environments and their dual-purpose economic value in wool and meat production [13]. Due to their high-quality wool, wide adaptability across geographic regions, and economic importance in global production systems, Merino sheep have spread worldwide, both as purebreds and through hybridization.

Sheep husbandry constitutes an important livelihood for rural populations in Türkiye and serves as the cornerstone of small ruminant production within the national livestock sector [14,15]. Sheep are uniquely adapted to extensive steppe and rangeland ecosystems, owing to their physiological resilience to harsh climatic conditions and their ability to efficiently upcycle low-quality forage [16]. In addition to meat production, sheep farming contributes economically and environmentally by providing multiple products, including milk, wool, leather, and manure, which can be recycled as organic fertilizer [17]. Compared to other livestock sectors, the relatively low capital intensity of sheep farming makes it particularly prevalent in developing and transition economies [18]. However, the economic and environmental status of sheep wool has shifted markedly; in recent years, it has transitioned from a high-value commodity to a low-value by-product, or even a management challenge, due to declining global demand and escalating processing costs. [19,20].

The intensification of sheep farming, characterized by increased animal density and waste generation, may exert significant pressure on soil, water, and atmospheric systems if not properly managed [21]. To evaluate these environmental impacts, the Carbon Footprint (CF) has emerged as a key indicator, quantifying total direct and indirect GHG emissions—expressed as CO_2_ Equivalents—throughout the life cycle of a product or production system [22]. Various methodological frameworks, ranging from simplified estimation methods to advanced Life Cycle Assessment (LCA) and input–output-based approaches, have been developed to enable comprehensive environmental evaluations of agricultural systems. CF assessments are typically conducted using the methodological frameworks developed by the Intergovernmental Panel on Climate Change (IPCC), which categorize emission estimates into three levels—Tier 1, Tier 2, and Tier 3—based on data requirements and calculation complexity [23]. Tier 1 relies on default emission factors and generalized activity data, providing a simplified estimation approach. In contrast, Tier 2 incorporates country- or farm-specific parameters, such as animal performance, feed characteristics, and manure management practices, enabling more accurate, context-sensitive emission estimates. Tier 3 involves advanced process-based modeling and detailed datasets, offering the highest level of methodological precision. In this study, the Tier 2 methodology was specifically selected over Tier 1 to avoid the uncertainties associated with global default emission factors, which often fail to reflect the unique dietary and management characteristics of Mediterranean sheep systems. Furthermore, while Tier 3 provides higher precision, Tier 2 offers a more practical and applicable framework for regional policy development in Türkiye, where high-resolution farm-specific activity data are available but process-based modeling parameters are not yet fully standardized.

The present study was conducted to quantify the farm-level carbon footprint and to identify critical environmental hotspots within commercial sheep farming systems in the Bursa region of Türkiye. By applying the IPCC Tier 2 methodology across four commercial farms representing intensive and semi-intensive management practices, emissions were estimated within an expanded ‘cradle-to-farm-gate’ system boundary. In contrast to assessments limited to biogenic processes, the environmental burden associated with feed production and on-farm energy use was incorporated to provide a comprehensive life-cycle perspective. Furthermore, beyond establishing a comparative baseline, specific and actionable mitigation strategies, centered on supply chain optimization and manure management, are identified to enhance the operational sustainability of the livestock sector.

## 2. Materials and Methods

### 2.1. Study Area and Farm Characterization

The study was conducted across four commercial sheep farms situated in the northern part of Bursa, Türkiye. While the southern and inland territories of the province exhibit a continental climate, the northern region is characterized by a Mediterranean climate. The environmental and climatic parameters of the study area are summarized in Table 1 [24].

These farms were selected as representative models of commercial Merino sheep enterprises operating under management conditions (Table 2). The study flocks consisted of adult Merino sheep (mature ewes and breeding rams) that had attained physiological maturity and target slaughter weight (60.0 ± 1.8 kg, mean ± SE). The animals were housed in segregated compartments within the facilities. The assessment focused exclusively on the adult flock, representing the primary and most stable production unit. Lambs were intentionally excluded from the system boundaries due to their seasonal presence, distinct physiological requirements, and relatively marginal contribution to the total annual farm-level greenhouse gas emissions.

The sheep were housed in confined barns provided with bedding materials consisting of a mixture of straw and wooden chips. In accordance with routine farm management practices, manure was allowed to accumulate on the bedding and was removed from the facilities at six-month intervals. The housing units were naturally ventilated to facilitate continuous gaseous exchange between the indoor and outdoor environments.

The feed industry is highly developed in the region where the farms are located. Consequently, farmers tend to rely on commercially manufactured feeds provided by large-scale feed companies rather than producing their own rations, due to their consistency, reliability, and practical advantages. In the farms included in this study, concentrate feeds were predominantly used, reflecting a production strategy aimed at maximizing growth performance and overall productivity. The detailed nutritional composition and analytical constituents of the diet are presented in Table 3. Throughout the study period, the sheep had ad libitum access to drinking water.

Despite the similarities in feed composition and breed, the classification of the farms (Table 2) is based on operational intensity and resource management. The intensive farms (SF2 and SF4) operate as high-throughput units with higher stocking densities, automated systems, and a strictly controlled indoor environment to maximize growth per unit of time. In contrast, the semi-intensive farms (SF1 and SF3) follow a more traditional, low-input management style. In these systems, while the same commercial feeds are used, the daily routine involves lower animal density per square meter and a less mechanized approach to manure and feed handling, reflecting the regional transition between traditional and industrial production models.

### 2.2. System Boundaries and Scope of the Analysis

**Figure 1:**
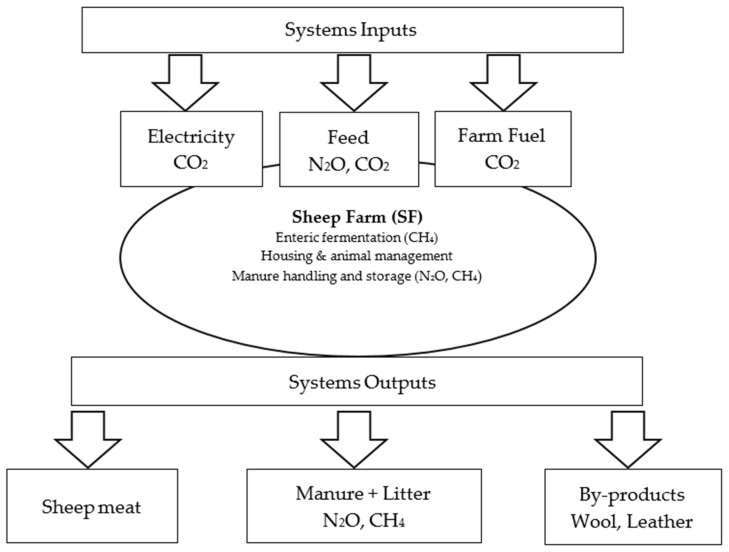
Farm-gate system boundaries of the monitored sheep production systems.

The system boundaries for this assessment encompass all processes from the acquisition of raw materials to the farm-gate exit, adopting a comprehensive ‘cradle-to-gate’ approach. All relevant inputs and outputs integrated within these boundaries are illustrated in Figure 1. System inputs include electricity and fuel consumption, along with the production of feed resources used in sheep farming, while outputs comprise sheep meat, manure, and litter. Although the monitored farms do not possess agricultural land or engage in on-farm crop cultivation, the emissions associated with externally sourced feed (roughage and concentrates) were explicitly integrated into the system boundaries. Recognizing that feed production is a significant upstream contributor to GHG emissions in ruminant systems, these indirect loads were quantified using regional life-cycle emission factors. This integration ensures a more accurate representation of the total environmental impact, accounting for the entire supply chain required to sustain intensive sheep production.

Furthermore, sheep wool was treated as a by-product and excluded from the system boundary. This decision was based on its negligible economic significance within the studied enterprises, where production is primarily oriented toward meat yield, and the market value of wool often fails to cover shearing and handling costs. Considering the prevailing market conditions and operational practices of the monitored farms, wool contributes less than 1% to the total revenue. Consequently, following the ISO 14044 cut-off rule, wool was excluded from the system boundary as its inclusion would have a negligible impact on the final results, and all environmental burdens were allocated to meat production as the primary economic driver [25].

The primary sources of GHG emissions in sheep production systems encompass enteric fermentation, manure and litter management, feed production, and on-farm energy consumption (fossil fuel combustion and electricity). Within the established system boundaries, GHG emissions were categorized into three distinct groups: (i) emissions arising from biological production processes, specifically enteric fermentation and manure management; (ii) indirect emissions associated with the production of dietary requirements (roughage and concentrates); and (iii) emissions resulting from on-farm energy consumption, including electricity and fuel use.

One limitation of this study is its regional focus on four commercial farms in Bursa, Türkiye; therefore, the findings reflect the specific characteristics of the local feed industry and management practices. Additionally, the assessment was limited to the adult flock (ewes and rams), excluding other production stages. Future research incorporating the entire production cycle, including lamb growth, as well as studies conducted across different geographical regions, would provide a more comprehensive understanding of the national carbon footprint of sheep production.

#### 2.2.1. Emissions from Biological Production Processes

In this study, GHG emissions were quantified using a deterministic calculation-based approach. Emissions associated with enteric fermentation and manure management were estimated following the IPCC Tier 2 methodology, which relies on detailed animal energy requirements and nitrogen excretion pathways. Direct measurement of enteric CH_4_ emissions under field conditions is technically demanding and necessitates specialized instrumentation for long-term monitoring, the IPCC-recommended Tier 2 methodology was adopted as a robust alternative. Calculations were conducted in accordance with the 2006 IPCC Guidelines for National Greenhouse Gas Inventories, incorporating methodological refinements and updated emission factors from the 2019 IPCC Refinement where applicable.

Following the Tier 2 framework, CH_4_ estimations were based on net energy requirements and the dietary energy supply, utilizing standardized equations specific to sheep (Table 4) [26]. The net energy requirements accounted for maintenance, growth, physical activity, milk and wool production, and pregnancy. These energy-related parameters, alongside feed digestibility and locally relevant input data, were incorporated into the emission models. Parameters for estimating emissions from manure management systems were selected according to IPCC guidelines, specifically calibrated to reflect the climatic and management conditions of Türkiye.

The Tier 2 methodology necessitates the integration of country-specific parameters and farm-level activity data. In the present study, enteric CH_4_ emissions were quantified by calculating specific emission factors (EF) for each sheep farm (Table 5). Beyond enteric fermentation, manure management was identified as another significant source of CH_4_ emissions. CH_4_ is generated during the anaerobic decomposition of organic matter in manure management systems. The maximum CH_4_-producing capacity of the manure (B_0_) was determined based on daily volatile solids (VS) excretion. For this parameter, the IPCC default value for the Eastern Europe region was adopted, as it is considered representative of the livestock production systems and climatic conditions of the study area.

Furthermore, N_2_O emissions were calculated using the standardized equations specified in the IPCC Tier 2 methodology (Table 6). N_2_O generation in livestock systems originates either directly or indirectly from manure management within paddock and housing environments. In this study, the monitored farms employed paddock-based systems and solid-manure management practices. Direct N_2_O emissions are primarily governed by storage duration and the specific manure management techniques employed. Conversely, indirect emissions occur via nitrogen leaching or the volatilization of nitrogenous compounds, specifically in the form of ammonia (NH_3_) and nitrogen oxides (NO_x_).

#### 2.2.2. Emissions from Feed Production

In the sheep farms monitored in this study, the feed consists of concentrate and roughage. To meet the nutritional requirements for livestock maintenance, the daily feed requirement is calculated as a dry matter intake of 2.5% of the animal’s body weight [27]. Feed production involves various inputs, including seeds, pesticides, tractor use for tillage, harvesting, and fuel consumption throughout the entire production cycle.

To accurately reflect the environmental burden of the feed supply chain, a partitioned approach was adopted to account for the distinct nutritional requirements of different livestock categories. The total annual feed quantity (*Q_y_*) for each farm was calculated by distinguishing between ewes and rams (Equation (1)):

(1) $$Q_{y}=(N_{\mathrm{ewes}}\cdot D_{\mathrm{ewes}})+(N_{\mathrm{rams}}\cdot D_{\mathrm{rams}})$$

where *N_ewes_* and *N_rams_* denote the number of mature ewes and breeding rams, respectively, and *D_ewes_* and *D_rams_* represent their specific annual feed intake levels. Subsequently, the CO_2_ emissions originating from feed production (*EF_F_*) were calculated using Equation (2) [28]:

(2) $${EF}_{F}=\sum {(Q}_{y}\cdot q_{i}\cdot e_{f})$$

In this formula, *EF_F_* represents the CO_2_ emissions from the production of feed resources; *Qy* is the annual feed quantity derived from Equation (1); *q_i_* indicates the ratio of wheat, barley, maize, and sunflower within the feed composition; and *e_f_* is the CO_2_ equivalent emission factor for the respective feed types. In the sheep farms monitored for this study, the feed composition consists of 40% wheat, 30.18% barley, 12.0% maize, and 16.20% sunflower.

The emission factors for each feed component used to determine emissions originating from feed production were compiled from studies previously conducted in the region (Table 7). Roughage (wheat) accounts for 40% of the feed content, while 58.38% consists of concentrate feeds (barley, maize, and sunflower). Due to their negligible share in the ration, limestone, salt, and vitamins were omitted from the calculations.

#### 2.2.3. Emissions from On-Farm Energy Consumption

Greenhouse gas emissions associated with energy consumption were quantified by accounting for electricity use and fossil fuel combustion. Electricity consumption primarily encompassed lighting, ventilation systems, and the operation of ancillary farm equipment, while fuel consumption was attributed to the operation of feed distribution machinery. Emission estimations were based on primary energy and fuel consumption records obtained directly from the monitored farms, integrated with standardized emission factors from the literature.

For electricity consumption, an emission factor of 0.515 kg CO_2_-eq kWh^−1^ was applied. Emissions from diesel fuel consumption were calculated using a specific emission factor of 3.67 kg CO_2_-eq kg^−1^, consistent with established methodologies [31,32].

### 2.3. Reference Unit for Carbon Footprint Calculation

To ensure a standardized evaluation of climate impacts, CH_4_ and N_2_O emissions were converted into carbon dioxide equivalents. Consistent with the IPCC Fourth Assessment Report (AR4) guidelines, 100-year global warming potential (GWP_100_) values of 25 for CH_4_ and 298 for N_2_O were adopted.

In this study, the carbon footprint was calculated based on a specific reference unit. In alignment with established greenhouse gas accounting frameworks, a mass-based approach is frequently utilized to report emissions relative to the product output [33,34,35,36]. Accordingly, one kilogram of sheep meat (live weight) was adopted as the reference unit for all carbon footprint calculations in the present analysis.

## 3. Results

### 3.1. Gross Energy Requirement and GHG Emissions

The daily gross energy (GE) requirement of an individual sheep was estimated at approximately 23.1 MJ day^−1^ across all monitored farms. Given that the sheep breed, feeding regimes, and management practices were consistent among the facilities, a uniform daily energy requirement per animal was applied This consistency across farms is attributed to the standardized management practices and the use of uniform IPCC Tier 2 coefficients for the specific sheep categories under study. Accordingly, the cumulative daily energy requirements for the entire flocks were calculated as 4.6, 11.5, 3.5, and 18.5 GJ day^−1^ for SF1, SF2, SF3, and SF4, respectively. While these cumulative values scale with flock size, the energy requirement expressed per reference unit remained constant at 0.92 MJ kg^−1^ sheep meat across all farms (Supplementary Materials).

The CH_4_ emission factor associated with enteric fermentation (EF_E_) was estimated at 10.15 kg CH_4_ head^−1^ year^−1^. Based on this factor, total annual CH_4_ emissions were 2030, 5074, 1522, and 8118 kg CH_4_ year^−1^ for SF1, SF2, SF3, and SF4, respectively. While these total emissions scaled with the inventory size—placing SF4 as the largest contributor in absolute terms—the emission intensity per unit of product remained consistent across all facilities. Relative to the reference unit, enteric fermentation contributed 0.41 kg CH_4_ kg sheep meat^−1^. This consistency reflects the standardized nutritional management and physiological homogeneity of the Merino flocks in the region, indicating that while the scale of production differs, the biological efficiency of methane production relative to meat output is similar across the monitored systems. Consistent with enteric emissions, the manure-related climate impact was directly proportional to the farm inventory, maintaining a stable environmental load per kilogram of meat produced.

CH_4_ emissions from manure management totaled 3.92 kg CH_4_ head^−1^ year^−1^. When normalized to the reference unit, this corresponded to 0.16 kg CH_4_ kg^−1^ sheep meat. Based on flock size, the total annual CH_4_ emissions from manure management were estimated at 784, 1960, 588, and 3137 kg CH_4_ year^−1^ for SF1, SF2, SF3, and SF4, respectively.

In the monitored farms, manure was stored as solid waste on straw bedding, while the animals maintained free access between indoor and outdoor areas. Consequently, a portion of the manure was deposited on paddock surfaces, facilitating nitrogen infiltration into the soil and contributing to N_2_O emissions. The calculated direct N_2_O emissions from manure management were 14.5, 36.2, 10.9, and 57.9 kg N_2_O year^−1^ for SF1, SF2, SF3, and SF4, respectively. Indirect N_2_O emissions were estimated at 10.9, 27.1, 8.1, and 43.4 kg N_2_O year^−1^. Relative to the reference unit, direct and indirect N_2_O emissions corresponded to 2.89 × 10^−3^ and 2.17 × 10^−3^ kg N_2_O kg^−1^ sheep meat, respectively. Furthermore, the annual direct and indirect N_2_O emissions per animal were determined to be 0.072 kg N_2_O and 0.054 kg N_2_O, respectively.

### 3.2. CO_2_ Emissions from Feed Production

To provide a comprehensive assessment of emissions originating from sheep production, CO_2_ emissions related to feed components were also analyzed within the system boundaries. EF_F_ values were calculated using emission factors that account for a daily dry matter intake (DMI) of 1.5 kg per mature ewe and 2.0 kg per ram, the specific contents of the feed rations used on the farms.

**Figure 2:**
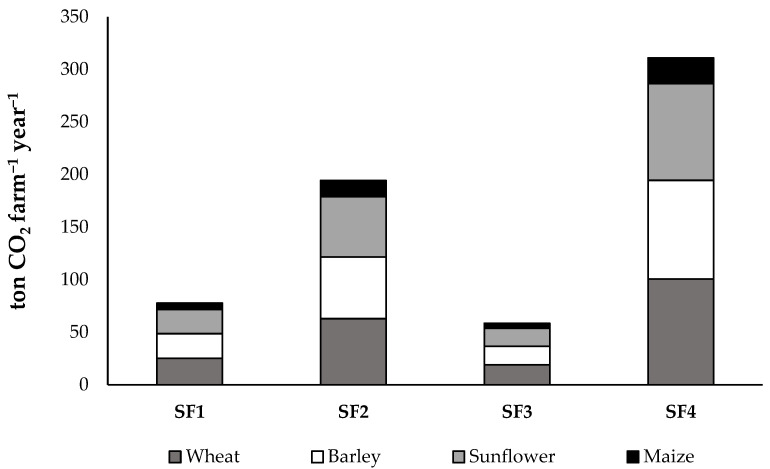
CO_2_ emissions from on-farm feed production in the monitored sheep farms.

The analysis revealed that the daily feed-related emissions differ significantly between livestock categories; while a single mature ewe results in 0.705 kg CO_2_-eq day^−1^, a breeding ram accounts for 0.940 kg CO_2_-eq day^−1^. Annual CO_2_ emissions attributed solely to feed production showed significant variations among the four farms, reflecting both flock size and the differentiated nutritional requirements of ewes and rams (Figure 2). Consistent with its intensive feeding regime and high livestock population, SF4 recorded the highest feed-related emission load (310.8 tons CO_2_-eq year^−1^). In contrast, SF3, with the smallest flock size, exhibited the lowest annual carbon footprint related to feed consumption (58.4 tons CO_2_-eq year^−1^).

The analysis of feed-related emissions indicates that total emissions are linearly correlated with flock size; however, due to the standardized ration formulation, the internal distribution of environmental burdens remains consistent across all farms. While the daily dry matter intake was differentiated by animal category (1.5 kg for ewes and 2.0 kg for breeding rams), sunflower meal and wheat straw were identified as the primary environmental hotspots, accounting for 30.2% and 32.3% of the feed-related carbon footprint, respectively. Despite its lower inclusion rate in the total dry matter intake, the high emission intensity of sunflower meal alone underscores the significant impact of high-protein concentrates on the overall environmental sustainability of sheep production. Furthermore, barley contributed 29.6% to the total emissions, while maize represented the smallest environmental burden at 7.9%.

### 3.3. CO_2_ Emissions from Energy Consumption

Energy consumption in livestock operations is essential for maintaining environmental conditions and facilitating daily management tasks. Within the monitored farms, the primary energy sources identified were diesel fuel, utilized for feed distribution machinery, and electricity, used for facility lighting and ancillary systems.

The daily feed ration per animal and the operational duration of feed distribution machinery were standardized based on the common management protocols observed across the monitored farms. Consequently, emissions from diesel fuel consumption were determined to be 1.6 kg CO_2_-eq per kg of sheep meat. While the total annual emissions scaled with the flock size—totaling 19.2, 48.0, 14.4, and 76.8 tonne CO_2_ for SF1, SF2, SF3, and SF4, respectively—this uniformity reflects a consistent level of mechanization efficiency across the four enterprises. In this context, the higher total impact in SF4 is a direct consequence of the larger operational volume handled under similar technological conditions.

The average emission from electrical units per reference unit across the monitored farms was estimated at 0.036 kg CO_2_-eq. The annual electricity-related emissions were 598.34 kg CO_2_ (SF1), 842.73 kg CO_2_ (SF2), 434.85 kg CO_2_ (SF3), and 869.84 kg CO_2_ (SF4). Notably, electricity consumption did not scale linearly with flock size as strictly as biogenic emissions. In SF4, which has the largest flock, electricity demand per animal is influenced by the facility’s structural design, requiring supplemental lighting during specific hours in deep-bedded indoor sections, regardless of the exact animal count (Supplementary Materials). This lack of strict linearity between inventory size and electricity use highlights that infrastructure efficiency, rather than just animal numbers, plays a role in the energy-related carbon footprint.

### 3.4. Total Carbon Footprint Analysis

**Figure 3:**
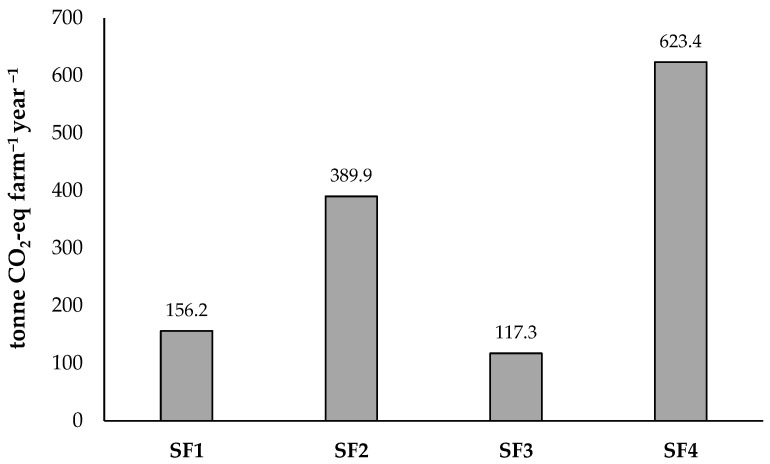
Total annual carbon footprint across the four monitored sheep farms (tonne CO_2_-eq farm^−1^).

The cumulative annual carbon footprint of the monitored sheep farms, integrating biological sources (methane and nitrous oxide), on-farm energy consumption (carbon dioxide), and feed production emissions, is summarized in Figure 3. In this framework, annual CH_4_ and N_2_O outputs were quantified utilizing the IPCC Tier 2 methodology, while CO_2_ emissions were derived from primary on-farm energy data and consumption for feed ingredients. Among the evaluated facilities, SF4 exhibited the most substantial total carbon footprint, whereas SF3 recorded the lowest; these variations were primarily attributed to differences in flock population and the corresponding scale of operations (Figure 3).

Focusing exclusively on biogenic emissions, specifically CH_4_ from enteric fermentation and CH_4_ and N_2_O from manure management, the carbon footprint was determined to be 15.6 kg CO_2_-eq per kg of sheep meat. However, when the system boundary was expanded to include indirect emissions from feed production and on-farm energy use, the average carbon footprint rose to an average of 28.8 kg CO_2_-eq for ewes and 32.3 kg CO_2_-eq for breeding rams per kg of boneless sheep meat. This expansion reveals that feed production is the most significant indirect contributor, accounting for approximately 49.8% of the total climate impact, while the relative share of on-farm energy consumption was found to be minor (0.29%) within the broadened system boundaries (Figure 3).

The relative contribution of various emission sources to the total carbon footprint exhibited a highly consistent structural pattern across both semi-intensive (SF1, SF3) and intensive (SF2, SF4) sheep farming systems. Within the production chain, feed production and enteric fermentation emerged as the two primary drivers for all examined systems. Specifically, feed production represented the largest share at 49.8% of the total footprint, followed by enteric fermentation at 32.5%. Emissions from manure management (manure + litter) accounted for 17.4%, while on-farm energy use represented a minor fraction of 0.29% of the cumulative emissions. This structural uniformity across both intensive and semi-intensive models underscores that the environmental burden is consistently dominated by nutritional and digestive processes in the region, regardless of the specific farming intensity or management scale. In contrast, emissions from manure management and energy-related activities remained secondary factors across all farming systems, emphasizing that regional mitigation strategies should prioritize nutritional efficiency to effectively lower the cumulative carbon footprint.

## 4. Discussion

In this study, the carbon footprint of four commercial sheep farms in the Bursa region was determined to assess their contribution to climate change. The results demonstrate that system boundaries significantly influence the final carbon footprint value. While biological emissions initially accounted for 15.6 kg CO_2_-eq, the most substantial increment occurred with the integration of indirect feed production, finalizing the cumulative carbon footprint at 28.8 kg CO_2_-eq for ewes and 32.3 kg CO_2_-eq for breeding rams. This indicates that in intensive and semi-intensive regional operations, indirect feed-related emissions and on-farm energy management collectively contribute approximately 49.8% of the total climate impact.

In alignment with previous research, the present findings confirm that enteric fermentation remains the primary driver of CH_4_ emissions in sheep production systems [37]. The enteric CH_4_ emission factor estimated in this study demonstrates strong consistency with existing local literature. For instance, Yıldırır et al. [38] reported emission factors of 8.55 kg CH_4_ head^−1^ year^−1^ for Anatolian Merino sheep and 10.86 kg CH_4_ head^−1^ year^−1^ for Hairy goats, derived from gross energy requirements of 21.7 MJ and 30.1 MJ head^−1^ day^−1^, respectively. The observed CF of 28.8 kg CO_2_-eq for ewes and 32.3 kg CO_2_-eq for breeding rams exceed some global ranges (8.9 to 17 kg CO_2_-eq) primarily due to our expanded boundaries. While studies often focus on biogenic-only assessments, our findings underscore that in intensive operations like SF4, mechanization and dietary inputs are non-negligible components of the environmental load.

The CF values obtained in this study, finalizing at 28.8 kg CO_2_-eq for ewes and 32.3 kg CO_2_-eq for breeding rams, exceed the typical global range of 8.9 to 17 kg CO_2_-eq reported for diverse sheep systems [33,38,39,40]. While our results are initially comparable to recorded for Merino sheep in Australia [41] when considering only on-farm activities, the inclusion of indirect emissions from feed production pushes our findings beyond these limits. This discrepancy is primarily attributed to our expanded ‘cradle-to-farm-gate’ boundary. The inherent variation in global literature is largely driven by these disparities in boundaries; many studies exclude the significant carbon burden of feed cultivation and concentrate processing, which in our case, accounted for a substantial portion of the final footprint.

The inclusion of both intensive (SF2, SF4) and semi-intensive (SF1, SF3) systems provides a nuanced understanding of how management intensity reshapes carbon footprints. Although standardized feeding regimes led to stable emission intensities across all farms, distinct disparities emerged in energy and manure-related outputs. Intensive systems (SF2, SF4) exhibited higher absolute energy-related emissions, driven by increased mechanization for feed distribution and continuous housing—a finding that confirms higher stocking densities necessitate greater energy inputs per animal unit.

The transition to a ‘cradle-to-farm-gate’ LCA further highlights these differences: while intensive systems are heavily skewed toward feed production and on-farm energy use, semi-intensive systems retain a higher relative contribution from enteric fermentation due to lower industrial energy reliance. So, mitigation strategies must be tailored to the production model: intensive farms should prioritize energy efficiency and concentrate selection, whereas semi-intensive systems may benefit more from grazing management and optimized nitrogen cycling to reduce their overall environmental load.

N_2_O represents a critical greenhouse gas in this assessment, primarily originating from nitrogen losses during manure handling and storage [42,43]. The elevated indirect N_2_O levels observed suggest significant nitrogen leaching and volatilization, likely driven by the prolonged six-month manure storage duration. As noted by Bell et al. [44], higher stocking densities in intensive systems often exacerbate these losses. Our findings emphasize that manure management is a vital lever for mitigation; implementing more frequent removal or improved storage covers could serve as a viable pathway to reduce the carbon footprint.

In conventional sheep systems, enteric fermentation typically accounts for 70–80% of total GHG emissions [45,46]. In the present study, the relatively lower share of enteric fermentation (32.5%) compared to these global averages is a direct result of our more comprehensive system boundary. This underscores the sensitivity of carbon accounting: as on-farm mechanization and upstream feed production are integrated, the proportional impact of biogenic sources is rebalanced. This finding suggests that mitigation strategies in intensive systems must be multi-dimensional, targeting feed efficiency and energy optimization alongside traditional nutritional interventions.

The relatively stable emission intensity per reference unit observed across the studied farms can be explained by the specific management characteristics of these systems. Standardized feeding regimes, uniform slaughter weights, and comparable housing and manure management structures contributed to similar per-animal energy requirements and emission factors. Furthermore, since the monitored flocks consisted predominantly of adult animals at similar body weights and physiological stages, structural disparities in flock composition were minimized. Consequently, variations in total farm emissions were primarily driven by flock size, whereas the emission intensity was influenced by operational efficiency rather than flock structure.

Regarding manure management, the six-month removal intervals likely exacerbated indirect N_2_O emissions through nitrogen volatilization and leaching. Higher stocking densities in intensive systems, intensify these losses. Consequently, mitigation strategies should prioritize optimizing manure storage conditions and enhancing feed efficiency rather than simply reducing flock size. The data suggests that a shift toward a 3-month manure removal cycle could potentially reduce indirect N_2_O loads by minimizing the anaerobic accumulation time.

From a practical perspective, our findings indicate that producers can mitigate emission intensity without compromising economic viability by improving operational efficiency. To support this transition, targeted policy actions are needed to motivate farmers to adopt more sustainable practices. For instance, the adoption of precision feeding strategies and local fodder sourcing, which addresses the 49.8% contribution from feed production, could be encouraged through regional subsidies or low-carbon certification schemes for meat products. Such certification could enhance product value and provide direct economic incentives for farmers.

Furthermore, governmental support in the form of low-interest green loans could facilitate the adoption of renewable energy technologies, such as photovoltaic systems. Although on-farm energy use represents a relatively small share (0.29%) of the total carbon footprint, transitioning to renewable energy is an important step toward net-zero targets and may reduce long-term operational costs, thereby offering both environmental and economic benefits.

These mitigation measures and policy approaches are also aligned with national strategies in Türkiye, including the Green Deal Action Plan and the National Climate Change Strategy, which emphasize energy efficiency, sustainable manure management, waste-to-energy conversion, and the development of low-carbon livestock production systems.

## 5. Conclusions

These findings demonstrate that indirect emissions from the feed supply chain represent the most significant source of the environmental load, accounting for 49.8% of the total climate impact. Consequently, producers can effectively mitigate emission intensity without reducing flock size by prioritizing feed conversion efficiency and optimized manure management. Specifically, shortening manure accumulation periods and improving storage conditions are critical interventions to reduce indirect nitrogen losses in intensive systems. Ultimately, this study underscores that greenhouse gas mitigation in the livestock sector must be addressed through a comprehensive ‘cradle-to-farm-gate’ Life Cycle Assessment (LCA) framework. Decarbonizing small ruminant production requires the synchronized management of all production stages—from feed cultivation to on-farm energy use and biogenic processes—treating these as an integrated lifecycle rather than isolated activities to achieve a holistic reduction in the overall carbon footprint.

Future research should focus on expanding this framework by incorporating the complete life cycle of the flock, particularly lamb growth phases and seasonal grazing dynamics, to provide a more granular temporal and spatial analysis. Additionally, the integration of digital monitoring systems for real-time emission tracking, along with the evaluation of the long-term effects of locally available agricultural waste additives, could further refine mitigation strategies. Furthermore, combining environmental assessments with economic analyses would provide a more robust basis for developing sustainable, scalable, and economically viable strategies for reducing emissions in sheep production systems.

## Figures and Tables

**Table 1:** Long-term meteorological characteristics of the study area.

Mean Temp. (°C)	Mean Max. Temp. (°C)	Mean Min. Temp. (°C)	Total Precipitation (mm)	Mean Rainy Days	Mean Sunshine Duration (h)
14.7	20.5	9.2	706.4	111.5	6.2

**Table 2:** General characteristics of the monitored sheep farms.

	Sheep Farm 1 (SF1)	Sheep Farm 2 (SF2)	Sheep Farm 3 (SF3)	Sheep Farm 4 (SF4)
Number of sheep (head)	200	500	150	800
Production system	Semi-intensive	Intensive	Semi-intensive	Intensive
Manure management	Solid storage/range	Solid storage/range	Solid storage/range	Solid storage/range

**Table 3:** Composition and nutritional value of the diet.

**Ingredients (% of DM)**	
Ingredient	% of DM
Wheat Straw	40.0
Barley	30.18
Maize	12.00
Sunflower seed meal	16.20
Limestone	0.90
Salt	0.60
Vitamin-Mineral Premix	0.12
**Chemical Composition of the diet**	
Parameter	Value
Crude Protein (%)	14.0
Crude Fiber (%)	8–10
Crude Ash (%)	10.0
Sodium (%)	0.25
Crude Fat (%)	3–4.5
Vitamin A (IU/kg)	9000
Vitamin D3 (IU/kg)	3000
Vitamin E (mg/kg)	25
DM: Dry Matter	

**Table 4:** IPCC Tier 2 equations utilized for estimating the net energy requirements of sheep [26].

Type of Metabolic Functions	Equations	IPCC Equation No
Net energy for maintenance (MJ day^−1^)	NE_m_ = C*f_i_* · Weight^0.75^	Equation 10.3
Net energy for animal activity (MJ day^−1^)	NE_a_ = C_a_ · Weight	Equation 10.5
Net energy needed for growth (MJ day^−1^)	NE_g_ = $\frac{{WG}_{lamb/kid}\cdot (a+0.5b(BWİ+BWf)}{365}$	Equation 10.7
Net energy for lactation for sheep (MJ day^−1^)	NE_l_= $\left[\frac{\left(5\cdot {WG}_{wean}\right)}{365}\right]$·EV_milk_	Equation 10.9
Net energy required to produce wool (MJ day^−1^)	NE_wool_ = $\left(\frac{{EV}_{wool}\cdot {Pr}_{wool}}{365}\right)$	Equation 10.12
Net energy required for pregnancy (MJ day^−1^)	NE_P_ = C_pregnancy_ · NE_m_	Equation 10.13
Ratio of net energy available in a diet for maintenance to digestible energy	REM = [1.123 − (4.092 · 10^−3^ · DE) + (1.126 · 10^−5^ · (DE)^2^) − (25.4/DE)]	Equation 10.14
Ratio of net energy available for growth in a diet to digestible energy consumed	REG = [1.164 − (5.16 · 10^−3^ · DE) + (1.308 · 10^−5^ · (DE)^2^) − (37.4/DE)]	Equation 10.15
Gross energy for sheep (MJ day^−1^)	$GE=\left[\frac{\left(\frac{{NE}_{m}+{NE}_{a}+{NE}_{l}+{NE}_{p}}{REM}\right)+\left(\frac{{NE}_{g}+{NE}_{wool}}{REG}\right)}{DE}\right]$	Equation 10.16

C_fi_ is the coefficient (0.217 for sheep > 1 year and 0.236 for lambs < 1 year, MJ day^−1^ kg^−1^); C_a_ is the activity coefficient (0.0067 for housed fattening sheep, MJ day^−1^ kg^−1^); a and b are constants for NE_g_ calculation (a = 2.5, b = 0.35 for intact males; a = 2.1, b = 0.45 for females, MJ kg^−1^); BW_i_ and BW_f_ represent the live body weight at weaning and at 1 year old (or slaughter), respectively (kg). WG_lamb/kid_ is the annual weight gain (kg year^−1^), and WG_wean_ is the weight gain between birth and weaning (kg). EV_milk_ and EV_wool_ denote the energy required to produce 1 kg of milk and the energy value of wool (MJ kg^−1^), while Pr_wool_ is the annual wool production per animal (kg year^−1^). C_pregnancy_ is the pregnancy coefficient (0.077 for single birth), and DE is the digestibility of feed expressed as a fraction of gross energy.

**Table 5:** Tier 2 equations for calculating enteric and manure-related CH_4_ emission factors [26].

Type of Metabolic Functions		Equations		IPCC Equation No	
Emission factors from enteric fermentation (kg CH_4_ head^−1^ year^−1^)		EF_E_ = $\frac{GE\cdot \left(\frac{Y_{m}}{100}\right)\cdot 365}{55.65}$		Equation 10.21	
Emission factor from manure management (kg CH_4_ head^−1^ year^−1^)		EF_M_ = (VS · 365)$\left[B_{0}\cdot 0.67\cdot \sum \frac{MCF}{100}\cdot AWMS\right]$		Equation 10.23	
Volatile solid excretion rates (kg VS day^−1^)		VS = $\left[GE\cdot \left(1-\frac{DE}{100}\right)+(UE\cdot GE)\right]$·$\left[\left(\frac{1-ASH}{18.45}\right)\right]$		Equation 10.24	
CH_4_ emissions from manure management (Gg CH_4_ year^−1^)		CH_4Manure_ = $\sum EF\cdot \left(\frac{N}{10^{6}}\right)$		Equation 10.19	
Y_m_: CH_4_ conversion factor, per cent of gross energy in feed converted to CH_4_ (6.7% ± 0.9); 6.7% was used in the calculations. B_o_: maximum CH_4_-producing capacity for manure produced by the livestock category, m^3^ CH_4_ kg^−1^ of VS excreted (0.19 m^3^ CH_4_ kg^−1^ VS) MCF: CH_4_ conversion factors for each manure management system by climate region, %			AWMS: fraction of livestock category UE·GE: urinary energy expressed as a fraction of GE, typically 0.04 GE can be considered urinary energy excretion by most ruminants ASH: the ash content of manure calculated as a fraction of the dry matter feed intake		

Y_m_ is the CH_4_ conversion factor, representing the percentage of gross energy in feed converted to CH_4_ (6.7% was used in the calculations); Bo is the maximum CH_4_-producing capacity for manure (0.19 m^3^ CH_4_ kg^−1^ of VS excreted). MCF denotes the CH_4_ conversion factors for each manure management system by climate region (%); AWMS is the fraction of livestock category managed by each system. UE·GE represents urinary energy expressed as a fraction of gross energy (typically 0.04 GE for most ruminants), and ASH is the ash content of manure calculated as a fraction of the dry matter feed intake.

**Table 6:** IPCC Tier 2 equations employed for the estimation of direct and indirect N_2_O emissions in sheep production systems [26].

**Direct N_2_O Emissions Equation**		**IPCC Equation No**
Direct N_2_O emissions from manure management (kg N_2_O year^−1^)	$N_{2}O_{D}=\left[\sum \left[\sum \left(N\cdot N_{ex}\right)\cdot AWMS)+N_{cdg}\right]\cdot {EF}_{3}\right]\cdot \frac{44}{28}$	Equation 10.25
**Indirect N_2_O Emissions Equations**		
The amount of manure nitrogen that is lost due to volatilization of NH_3_ and NO_x_, kg N year^−1^	$N_{volatilization-MMS}=\left[\sum \left[\sum (\left(N\cdot N_{ex}\right)\cdot AWMS)+N_{cdg}\right]\cdot {Frac}_{GasMS}\right]$	Equation 10.26
The amount of manure nitrogen that is lost due to leaching (kg N year^−1^)	$N_{leaching-MMS}=\left[\sum \left[\sum (N\cdot N_{ex}\cdot AWMS)+N_{cdg}\right]\cdot {Frac}_{LeachMS}\right]$	Equation 10.27
Indirect N_2_O emissions due to volatilization of N from Manure Management in the country, kg N_2_O year^−1^	N_2_O_G_ (mm) = (N_volatilization-MMS_ · EF_4_) · $\frac{44}{28}$	Equation 10.28
Indirect N_2_O emissions due to leaching and runoff from manure management (kg N_2_O year^−1^)	N_2_O_L_ (mm) = (N_leaching-MMS_ · EF_5_) · $\frac{44}{28}$	Equation 10.29
Annual N excretion rates (kg N head^−1^year^−1^)	N_ex =_ (N_intake_ − N_retention_) · 365	Equation 10.31A
N intake rates (kg N animal^−1^day^−1^)	N_intake_ = $\frac{GE}{18.45}\cdot \left(\frac{\frac{CP\%}{100}}{6.25}\right)$	Equation 10.32
Managed manure N available for application to managed soils, feed, fuel, or construction uses (kg N year^−1^)	$N_{MMS\_Avb}=\sum \left\{\sum \left[\begin{array}{c} \left(N\cdot Nex\cdot AWMS+N_{cdg}\right)\cdot (1-{Frac}_{LossMS})]+\\ \left[N\cdot AWMS\cdot N_{\mathrm{beddingMS}}\right] \end{array}\right]\right\}$	Equation 10.34

N_cdg_ is the annual nitrogen input via co-digestate (set to zero as no anaerobic digestion system was implemented). EF_3_, EF_4_, and EF_5_ are the emission factors for direct N_2_O emissions from manure management (0.10 kg N_2_O–N kg N^−1^), atmospheric deposition (0.010 kg N_2_O–N kg^−1^), and nitrogen leaching/runoff (0.0075 kg N_2_O–N kg^−1^), respectively. The factor 44/28 is used to convert N_2_O–N emissions to N_2_O. FracgasMS is the fraction of managed nitrogen that volatilizes (0.05), FracleachMS is the percentage of nitrogen losses due to runoff and leaching (1%), and Frac_LossMS_ is the total nitrogen lost in the manure management system. N_beddingMS_ represents the nitrogen from bedding. Diet-related factors include 18.45 (conversion for dietary GE per kg of dry matter), CP% (percent crude protein), and 6.25 (conversion from dietary protein to nitrogen). N_retention_ is the fraction of annual N intake retained by the animal (0.10 kg N animal^−1^ day^−1^).

**Table 7:** Daily dry matter intake and associated carbon footprint of feed ingredients.

Nutrient Composition	Daily Feed Consumption (Ewes) (kg DM head^−1^)	Daily Feed Consumption (Rams) (kg DM head^−1^)	Emission Factor (kgCO_2_-eq kg^−1^)	References
Wheat	0.60	0.80	0.38	Taş et al. [29]
Barley	0.45	0.60	0.46	Taş et al. [29]
Maize	0.18	0.24	0.31	Taş et al. [29]
Sunflower seed meal	0.24	0.32	0.875	Yousefi et al. [30]

## Data Availability

The raw data supporting the conclusions of this article will be made available by the authors on request.

## Supplementary Materials

The following supporting information can be downloaded at: https://www.mdpi.com/article/10.3390/ani16071099/s1, Table S1: Lighting schedule and operational details for SF4; Table S2: Descriptive statistics and One-Way ANOVA results for gross energy estimations of four sampled farms.
