# Supplementary material for: Quantifying Greenhouse Gas Emissions and Carbon Footprint of Sheep Production Using the IPCC Tier 2 Approach
Source: Animals (Basel). 2026 Apr 2;16(7):1099. doi: 10.3390/ani16071099 (PMC13072365; doi:10.3390/ani16071099)
Supplement: Supplementary file 1 [file animals-16-01099-s001.zip › animals-4227735-supplementary.pdf]

Table S1. Lighting schedule and operational details for SF4.

Time Period	Lighting Source	Operational Status	Energy Dependency
07:00 – 11:30	Artificial (LED)	Manually switched on for morning feeding	Fixed
11:30 – 16:30	Natural + Limited Artificial	Supplemented during cloudy days or for inspection	Variable
16:30 – 20:30	Artificial (LED)	Manually switched on for evening management	Fixed
20:30 – 07:00	None	Lights switched off for rest period	None

Table S2. Descriptive statistics and One-Way ANOVA results for gross energy estimations of four sampled farms.

	Mean	Std. Deviation	Std. Error	95% Confidence Interval for Mean		Minimum	Maximum	Sig.
				Lower Bound	Upper Bound			
SF1	23.007[a]	0.09370	0.0296	22.940	23.074	22.88	23.15	0.566
SF2	23.100[a]	0.05249	0.0166	23.062	23.138	23.00	23.18	
SF3	23.007[a]	0.030576	0.0967	22.788	23.226	22.15	23.20	
SF4	23.030[a]	0.08869	0.08869	22.967	23.094	22.88	23.18	

[a]No statistically significant difference was observed between groups ($p > 0.05$).
